# Diagnostic Accuracy of Alpha-Methylacyl-CoA Racemase Immunohistochemical Expression for the Diagnosis of Ovarian and Endometrial Clear Cell Carcinomas

**DOI:** 10.30699/IJP.2023.556417.2925

**Published:** 2023-03-23

**Authors:** Fatemeh Nili, Soheib Fathi, Mansoureh Tavakoli, Elham Mirzaian, Maryam Lotfi

**Affiliations:** 1 *Department of Pathology, Cancer Institute, Imam Khomeini Hospital Complex, Tehran University of Medical Sciences, Tehran, Iran*; 2 *Department of Pathology, Shariati Hospital, Tehran University of Medical Sciences, Tehran, Iran*; 3 *Department of Pathology, Amir-Alam Hospital, Tehran University of Medical Sciences, Tehran, Iran*

**Keywords:** Accuracy, AMACR, Clear cell carcinoma, ovary, Endometrium, Immunohistochemistry

## Abstract

**Background & Objective:**

Clear cell carcinoma (CCC) is an uncommon histopathologic subtype of ovarian and endometrial carcinoma. Due to the morphologic overlapping with other subtypes of ovarian and endometrial carcinomas, an accurate diagnosis is crucial.

**Methods:**

In this study, 31 cases of ovarian clear cell carcinoma (OCCC), 28 endometrial clear cell carcinoma (ECCC), and 80 non-CCC subtypes (33 high-grade serous carcinomas of the ovary, 2 low-grade serous carcinomas, 10 ovarian endometrioid, 3 serous carcinomas and 29 endometrioid carcinomas of the endometrium) were investigated for immunohistochemical expression of AMACR. Sensitivity, specificity, positive predictive value (PPV), and negative predictive value (NPV) for the distinction of OCCC and ECCC from other histopathologic subtypes were calculated.

**Results:**

Positive AMACR staining was seen in 18 OCCCs (58%) and 10 ECCCs (35.7%). In the non-clear cell group, 44 cases of ovarian (98%) and 25 cases of endometrial carcinoma (78%) showed negative results. Only one case of ovarian endometrioid carcinoma and 7 cases (22%) of endometrial endometrioid carcinomas revealed a positive reaction (*P*<0.05). Collectively, sensitivity, specificity, PPV, and NPV of AMACR expression, for the diagnosis of OCCC were calculated as 58%, 98%, 94.7%, and 77.2%, respectively. The sensitivity, specificity, PPV, and NPV were shown to be as 35.7%, 78.1%, 58.8%, and 58.1%, respectively in the endometrium.

**Conclusion:**

AMACR may be a highly specific immunohistochemical marker for the distinction of serous and clear cell carcinoma. A small percentage of endometrioid carcinoma may show positive staining. The sensitivity of this marker may not be higher than the other well-known Napsin-A IHC marker.

## Introduction

Clear cell carcinoma (CCC) is an uncommon histopathologic subtype of ovarian and endometrial carcinomas (1). It accounts for about 10-12% of ovarian and 1-6% of endometrial carcinomas (2) (3). In the ovary, they usually present in stage I, but endometrial cancers are in higher stages of the disease at the time of presentation. Unlike high-grade serous carcinoma, CCC is not sensitive to platinum-based chemotherapy drugs. So they have a poor prognosis, especially, when they are presenting in the advanced stages of the disease (1, 4, 5). 

Considering these therapeutic and prognostic challenges, accurate diagnosis is crucial. Both ovarian and endometrial CCC are characterized by a mixture of papillary, tubule-cystic, and solid growth patterns. The papillary structures are simple and non-branching with a round hyalinized stromal core. The papillary and tubulo-cystic structures are lined by a single layer of cuboidal cells with clear or eosinophilic cytoplasm, large monomorphic nuclei with round or angulated contours, and prominent nucleoli. Nuclear pleomorphism may be focal. Mitotic activity is typically low (2, 6, 7). 

β^6^, ^8^). 

Alpha-methylacyl-CoA racemase (AMACR) is a mitochondrial and peroxisomal enzyme, which is essential for lipid metabolism (9). The expression of this maker is widely used for the diagnosis of prostate adenocarcinoma and papillary renal cell carcinoma (10-13). In recent studies, its utility for the diagnosis of Gynecologic CCCs has been investigated. We aim to evaluate the accuracy of this marker for the differentiation of OCCC and ECCC from the other histopathologic subtypes.

## Material and Methods

In this retrospective case-control study, 59 cases of ovarian and endometrial clear cell carcinomas were included as case group and 80 non-clear cell carcinomas of the ovary and endometrium were selected as the control group. All of the CCC cases were reviewed by two expert Gynecologic pathologists and confirmed by the IHC study for Napsin-A. The study was approved by the ethical committee of the Tehran University of Medical Sciences. 

??

For detection, a Master polymer plus detection system (MAD-006237QK) was used. After counterstaining with Hematoxylin and mounting, the slides were examined under a light microscope and scored as follows: any moderate or severe staining in 1-5% of tumor cells: score 1, 6-50%; score 2, >50%: score 3.

×**.**

## Results

A total number of 139 cases including 59 CCCs (31 OCCC and 28 ECCC), 80 non-clear cell carcinomas (37 high-grade serous, 2 low-grade serous, and 41 endometrioid carcinomas) were analyzed. The mean age of patients with OCCC (49.9 years) was significantly lower than those with ECCC (61.7 years) (*P*= 0.00). There was no statistically significant difference between the mean age of patients with OCCC (49.9 years) and non-clear cell subtypes (53.5 years) (*P*=0.17). Patients with ECCC were older (61.7 years) than those with non-clear subtypes (54.2 years) (*P*=0.02). The difference between the tumor stage in OCCC, ECCC, and non-clear cell group was also statistically significant (*P*=0.00). The frequency of different histopathologic subtypes in the ovary and endometrium as well as the mean age, tumor size, and stage are shown in Table 1. 

**Table 1:** Frequency of histologic subtypes in ovary and endometrium, age of the patients, size and stage of the disease

Tumor site	Histologic subtype	Frequency	Age (meanSD)	Mean size	Stage (number of cases)
Ovary	Clear cell carcinoma	31 (40.8%)	49.911.2	11.25.9	I 17 II 2 III 5 IV 3
	Non-clear cell Carcinoma - High-grade serous - Low-grade serous - Endometrioid	33 (43.4%) 2 (2.6%) 10 (13.2%)	53.312.5	8.86	I 8 II 7 III 25 IV 1
Endometrium	Clear cell carcinoma	28 (46.7%)	61.713.1	4.92.9	I 7 II 6 III 3 IV 6
	Non-clear cell carcinoma - Serous carcinoma - Endometrioid carcinoma	3 (5%) 29 (48.3%)	54.212.5	5.13.6	I 18 II 4 III 6 IV 1

Positive AMACR staining was seen in 18 out of 31 OCCCs (58%) and 10 out of 28 ECCCs (35.7%). The percentage of tumor cell staining in the positive cases is shown in Table 2. In the non-clear cell group, 44 cases of ovarian carcinomas (98%) showed negative results. Only one case of endometrioid carcinoma revealed a positive reaction (2%). Twenty-five cases of endometrial non-clear cell subtype (78%) had negative results (*P*=0.00). The remaining 7 cases (22%) were all endometrioid subtypes and showed variable expression of AMACR (*P*<0.05).

In this way, positive AMACR staining is 58% sensitive and 98% specific for the diagnosis of OCCC. It has 94.7% PPV and 77.2% NPV. In the endometrium, the sensitivity, specificity, PPV, and NPV are 35.7%, 78.1%, 58.8%, and 58.1%, respectively. 

All of the 37 HGSC and 2 LGSC were negative for AMACR, so for this distinction: It is a 100% specific marker, although the sensitivity is moderate. Eight out of 33 endometrioid carcinomas were positive for AMACR, so the specificity is 75%.

**Table 2:** Frequency and score of AMACR staining in different subtypes of ovarian and endometrial CCC and non-CCC

Tumor site				AMACR percentage				Total
				0% (negative)	1-5% (score 1)	6-50% (score 2)	> 50% (score 3)	
Ovary	Tumor subtype	Endometrioid carcinoma		9 90.0%	0 0.0%	1 10.0%	0 0.0%	10 100.0%
		High grade Papillary serous carcinoma		33 100.0%	0 0.0%	0 0.0%	0 0.0%	33 100.0%
		Low grade Papillary serous carcinoma		2 100.0%	0 0.0%	0 0.0%	0 0.0%	2 100.0%
		Clear cell carcinoma		13 41.9%	9 29.0%	8 25.8%	1 3.2%	31 100.0%
	Total			57 75.0%	9 11.8%	9 11.8%	1 1.3%	76 100.0%
Endometrium	Tumor subtype	Endometrioid carcinoma		22 75.9%	5 17.2%	1 3.4%	1 3.4%	29 100.0%
		High grade Papillary serous carcinoma		3 100.0%	0 0.0%	0 0.0%	0 0.0%	3 100.0%
		Clear cell carcinoma		18 64.3%	2 7.1%	5 17.9%	3 10.7%	28 100.0%
	Total			43 71.7%	7 11.7%	6 10.0%	4 6.7%	60 100.0%
Total	Tumor subtype	Endometrioid carcinoma		31 79.5%	5 12.8%	2 5.1%	1 2.6%	39 100.0%
		High grade Papillary serous carcinoma		36 100.0%	0 0.0%	0 0.0%	0 0.0%	36 100.0%
		Low grade Papillary serous carcinoma		2 100.0%	0 0.0%	0 0.0%	0 0.0%	2 100.0%
		Clear cell carcinoma		31 52.5%	11 18.6%	13 22.0%	4 6.8%	59 100.0%
	Total			100 73.5%	16 11.8%	15 11.0%	5 3.7%	136 100.0%

**Fig. 1:**
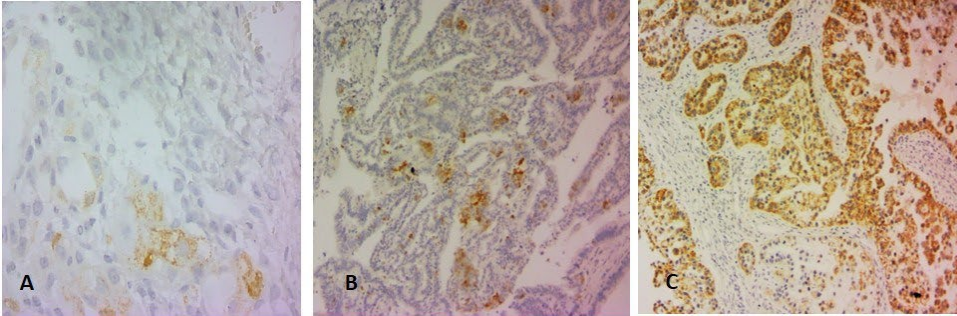
A) AMACR expression in 1-5% of tumor cells (score 1), B) 6-50% of the tumor cells (score 2), and C) more than 50% (score 3)

## Discussion

Histotyping of ovarian and endometrial carcinomas is a clinically significant subject, which potentially shows significant inter-observer variability in high-grade tumors (14, 15). Although most cases of CCC are diagnosed based on distinct morphological features, serous and endometrioid carcinoma with clear cell change or secretory features, closely mimic CCC (6, 13). 

In the present study, we evaluated the diagnostic accuracy of AMACR as a potential immunohistochemical marker for clear cell carcinoma histotype. The immunoreactivity of AMACR in ovarian clear cell carcinoma, endometrioid carcinoma, and serous carcinoma in the ovary was 58%, 10%, and 0%, respectively. In this way, the overall sensitivity of AMACR for the diagnosis of OCCC was 58.1%. The specificity, PPV and NPV were 97.8%, 94.7%, and 77.2%, respectively. In a 2015 study by Fadare* et al.*, the expression of AMACR, HNF1 β, and Napsin A markers was examined as diagnostic markers for ovarian CCC. In this study, which was performed on 279 ovarian tumors, the expression of HNF1β, Napsin A, and AMACR markers in 65 cases of CCC was obtained at 92%, 82%, and 63%, respectively. Also, the expression of HNF1β, Napsin A, and AMACR markers was reported in 101 cases of serous carcinoma 7%, 1%, and 1% and in endometrioid carcinoma 37%, 5.3%, and 0%. The sensitivity, specificity, PPV, and NPV of AMACR expression in the classification of cancer as clear cell histotype were 63%, 99%, 95% and 89%, respectively (16). The results in our study are comparable with findings in this study. We found a non-significant lower sensitivity than the previous study. Both studies show that AMACR is a highly specific IHC marker for differentiation of OCCCs from the other hitotypes. But the pathologists cannot rely on it as a sensitive marker. Among the primary ovarian carcinomas, HGSC is the most frequent malignancy. As it was expected, 73% (33/45) of ovarian non-clear cell carcinomas in our study, were HGSC. None of them show immune reaction with AMACR. Thus, the overall specifity of AMACR in OCCCs reached a high value. 

At the endometrium, the immunoreactivity of AMACR in clear cell carcinoma, endometrioid carcinoma, and serous carcinoma was 36%, 24%, and 0%, respectively. Therefore, the overall sensitivity, specificity, PPV, and NPV of AMACR were 35.7%, 78.1%, 58.8%, and 58.1%, respectively. A 2013 study by Oluwole Fadare* et al.*, which used AMACR to differentiate clear ECCC from endometrioid and serous carcinoma, found that AMACR expression in ECCC (75%) was significantly higher than endometrial serous carcinoma (15%) and endometrioid carcinoma of the endometrium (22%). The sensitivity and specificity, PPV, and NPV of AMACR expression in the classification of cancer as clear cell histotype were 75%, 79%, 74%, and 80%, respectively (17). The value of specificity in our study is very close to this study, but the sensitivity is much lower. The diagnosis of CCC in the endometrium is more challenging than in the ovary (18, 19). Accurate diagnosis depends on the experience of the pathologist and performing ancillary tests. In the endometrium, the most prevalent histologic type is endometrioid carcinoma. Twenty-four cases of endometrioid carcinomas in our study, and 22% of this histologic subtype in the aforementioned study by Oluwole Fadare *et al.* (17) showed positive reaction with AMACR. It means that AMACR is a lower specific marker for the distinction of endometrioid from CCC than serous carcinoma. The overall specificity of AMACR in ECCC is also lower than in OCCC, due to the higher prevalence of the endometrioid subtype. Squamous morular metaplasia with clear cell change is one of the mimickers of CCC. Arciuolo *et al.* reported positive diffuse and strong staining with AMACR in all 18 endometrioid carcinomas with squamous morular metaplasia (20). In 2019, Pors *et al.* examined the immunohistochemical expression of AMACR, Napsin A, and HNF1β in 18 Mesonephric and 55 endometrial/cervical carcinomas. They also found a 75% sensitivity for AMACR expression in endometrial/cervical CCCs. The specificity for distinction from Mesonephric carcinoma was 78% (21). 

The number of cases evaluated in each study, IHC techniques, and the inherent differences in the performance of primary and secondary antibodies can also influence the results in different studies.

The overall immunoreactivity of AMACR in both ovarian and endometrial clear cell carcinoma, endometrioid carcinoma, and serous carcinoma was generally 47.5%, 19.5%, and 0%, respectively. The overall sensitivity, specificity, PPV, and NPV of AMACR for differentiation of OCCC and ECCC from non-clear cell subtypes were 47.4%, 89.6%, 77.7%, and 69%, respectively. It is well known that AMACR is a positive marker in about 70% of papillary renal cell carcinomas, 71% of colorectal adenocarcinoma, and 77% of hepatocellular carcinomas (17, 22, 23). In the case of metastatic carcinoma, AMACR could not be a reliable marker for the distinction of female genital tract CCC.

Two other immunohistochemical markersreported in the literature have shown a relatively high association with CCC. One of these markers is Hepatocyte nuclear factor 1β (HNF1β), which was initially identified by analyzing the gene expression profile of OCCC, and subsequent validation studies of this marker revealed high sensitivity and specificity for this histotype (24). Similar findings in a small study of 33 endometrial carcinomas showed that all CCCs were positive for HNF1β and all non-CCCs were negative (25). However, subsequent studies have shown that 22-60% of endometrial serous carcinoma (at least focal), 5-35% of endometrial endometrioid carcinoma, a wide range of non-clear cell cervical cancers, and only 78-67% of uterine clear cell carcinomas are positive for HNF1β (26, 27).

Another marker for differentiating CCC from other histologic subtypes is aspartic peptidase "Napsin A". It is a marker with high sensitivity and high specificity for CCC. Sensitivity, specificity, PPV, and NPV are 88%, 98%, 98%, and 91% have been reported (28-31).

The small sample size of our study and the absence of ovarian mucinous carcinoma or other unusual subtypes of endometrial carcinoma are the main limitations of our study. Nonetheless, we investigated and compared both OCCC and ECC which were not performed in the previous studies.

## Conclusion

AMACR may be a highly specific immunohistochemical marker for the distinction of serous and clear cell carcinoma. Although it is more frequently expressed in CCC, a small percentage of endometrioid carcinoma may show positive staining. The sensitivity of this marker may not be higher than the other well-known Napsin-A IHC marker. A combination of IHC panels would be recommended to make an accurate diagnosis.

## Conflict of Interest

There is no conflict of interest to be disclosed.

## Funding

The study was funded by the Tehran University of Medical Sciences, with the gran number 99-3-101-42307.
